# Inequality in the distribution of Covid-19 vaccine: a systematic review

**DOI:** 10.1186/s12939-022-01729-x

**Published:** 2022-08-30

**Authors:** Mohsen Bayati, Rayehe Noroozi, Mohadeseh Ghanbari-Jahromi, Faride Sadat Jalali

**Affiliations:** 1 Health Human Resources Research Center, School of Management and Information Sciences, Shiraz University of Medical Sciences, Shiraz, Iran; 2 Health Human Resources Research Center, School of Health Management and Information Sciences, Shiraz University of Medical Sciences, Shiraz, Iran

**Keywords:** Covid-19, Vaccine, Inequality, Distribution, Economic

## Abstract

**Background:**

The equality in the distribution of vaccines between and within countries along with follow sanitation tips and observe social distance, are effective strategies to rid the world of COVID-19 pandemic. Inequality in the distribution of COVID-19 vaccine, in addition to causing inequity to the population health, has a significant impact on the process of economic recovery.

**Methods:**

All published original papers on the inequality of Covid-19 vaccine distribution and the factors affecting it were searched in PubMed, Web of Science, Scopus and ProQuest databases between December 2020 to 30 May 2022. Selection of articles, extraction of their data and qualitative assessment (by STROBE) were performed by two researchers separately. Data graphing form was used to extract detailed data from each study and then, the collected data were classified.

**Results:**

A total of 4623 articles were evaluated. After removing duplicates and screening the title, abstract and full text of articles, 22 articles were selected and entered into the study. Fifteen (68.17%) studies were conducted in the United States, three (13.64%) in Europe, three (13.64%) in Asia and one (6.66%) in Oceania. Factors affecting the inequality in the distribution of COVID-19 vaccine were classified into macro and micro levels determinants.

**Conclusion:**

Macro determinants of inequality in the Covid-19 vaccine distribution were consisted of economic (stability and country’s economic status, Gross Domestic Product (GDP) per capita, financial support and human development index), infrastructure and health system (appropriate information system, functional cold chains in vaccine transport, transport infrastructure, medical and non-medical facilities per capita, healthcare access and quality), legal and politics (vaccination allocation rules, health policies, political ideology and racial bias), and epidemiologic and demographic factors (Covid-19 incidence and deaths rate, life expectancy, vulnerability to Covid-19, working in medical setting, comorbidities, social vulnerability, incarceration and education index). Moreover, micro/ individual level factors were included in economic (household’s income, home ownership, employment, poverty, access to healthy food and residency in the deprived areas) and demographic and social characteristics (sex, age, race, ethnic, religion, disability, location (urban/rural) and insurance coverage).

**Supplementary Information:**

The online version contains supplementary material available at 10.1186/s12939-022-01729-x.

## Background

The SARS-CoV2 coronavirus first revealed in China in December 2019 [1]. On March 11, 2020, the World Health Organization (WHO) declared a severe outbreak of the acute respiratory virus coronavirus 2 (SARS-Covid-2) a pandemic, and the associated syndrome was named Coronavirus (Covid-19) [2]. Globally, the virus has spread to more than 200 countries [3]. According to WHO, as of May 27, 2022, there were 524,467,084 cases of COVID-19 and 6,285,171 deaths worldwide [4]. This disease has caused serious concerns about the general health of individuals [5]. People with certain health conditions, such as the elderly [6], chronic patients, people with severe obesity, chronic kidney disease, diabetes, arterial hypertension, and asthma [7], are more likely to develop Covid-19 [8]. The COVID-19 pandemic has led to the death and severe illness of many people, disruption of normal life, job loss, unhanding trade and shrinking national economies, especially in developing countries [9].

About 2 years after the outbreak of COVID-19, effective treatments for COVID-19 are constantly being updated. Some over-the-counter medications are prescribed and consumed without enough knowledge have been shown to be futile in reducing the risk of developing or treating COVID-19 [10]. These treatment approaches are more supportive and preventing transmission is the best way for public health [11]. The easiest way to deal with Covid-19 is to use a mask, follow sanitation tips and observe social distance [12]. According to the WHO, alongside public health and social distance, safe and effective vaccines are an important tool to protect people against COVID-19, save lives and reduce social disorders on a large scale. Moreover, equal access to COVID-19 [13]. So, an effective strategy to rid the world of this disease, with a significant reduction in the risk of infection and death due to COVID-19, is the nationwide distribution of vaccines in countries and within countries [14]. According to the WHO Vaccine equity campaign “Vaccine equity will accelerate the end of the pandemic. Achieving WHO’s vaccine equity targets will substantially increase population immunity globally, protect health systems, enable economies to fully restart, and reduce the risk of new variants emerging [15].”

A study by Bernal et al. (2021) found that a single dose of the BNT162b2 vaccine is around 60–70 effective in preventing symptomatic disease in adults aged 70 and older in the UK, and two doses are around 90–85% effective. People who have been vaccinated and experienced the symptoms are 44% less likely to be hospitalized and 51% are less likely to die than those who have not been vaccinated. Amit et al. (2021) estimated the effect of the vaccine at 85% on days 15 to 28 after the first dose, indicating that those who were vaccinated had less infection and symptoms of COVID-19 [16]. Since January 2020, when the first SARS-CoV-2 sequence became public, the scientific community has sought the rapid development of mRNAs, proteins, viral vectors, and other types of COVID-19 vaccines [17].

Due to the limited production of vaccines, priority is usually given in countries for their injection. The World Health Organization (WHO), the United States, and the United Kingdom prioritize health care personnel and people at high risk for serious complications and mortality, such as the elderly and people with comorbidities [18–20]. According to a study by Bubar et al. (2021), prioritizing vaccination for adults over 60 years of age with underlying disease is an appropriate strategy for reducing mortality from COVID-19 [21]. The results of a study by Buckner et al. (2020) showed that health care workers who are most at risk of infection and those over 60 years of age who are most at risk of dying from the disease are vaccinated [22]. Despite prioritizing vaccine, there is inequality in its distribution even within a group (e.g., health personnel. There are significant inequalities in COVID-19 burden in terms of race, ethnicity and socioeconomic status, which is influenced by the prioritization and distribution of vaccinations [23–26]. These inequalities are reduced when everyone has equal access to healthcare. For this reason, vaccine allocation strategies should reduce existing inequalities. Barriers to receiving the COVID-19 vaccine, including limited access to health care or living in rural and inaccessible areas, should also be identified and removed [27]. Inequality in the distribution of COVID-19 vaccine, in addition to causing injustice to the health of individuals and communities, has a significant impact on the process of economic recovery in developing countries. It was predicted that if the increase in vaccine production and distribution was sufficient for developing countries so that their vaccination rates were equal to those of developed countries, about $ 38 billion would be added to their Gross Domestic Product (GDP) in future year (https://www.who.int/news/item/22-07-2021-vaccine-inequity-undermining-global-economic-recovery). Despite the gradual reduction of inequality in vaccine distribution, the number of doses injected per population is 69 times higher in developed countries than in developing countries (https://www.thelancet.com/journals/laninf/article/PIIS1473-3099(21)00344-3/fulltext).

In addition to the importance of vaccination distribution, the willingness of individuals to receive Covid-19 vaccine is one of the major challenges in countries. However, the general desire for the Covid-19 vaccine is relatively high among the general population of the world. Nevertheless, skepticism is a major obstacle to global efforts to control the current pandemic [28]. Causes of this resistance may include safety concerns; in particular, the fear that the vaccine is dangerous because of its rapid production, plus the belief that the vaccine is useless; Due to the assumption that Covid-19 is harmless. Other reasons for public distrust, doubts about the effectiveness of the vaccine, belief in pre-existing immunity and doubts about the origin of the vaccine are other reasons [29]. According to a study by Sallam et al., The willingness to receive the vaccine in Kuwait, Saudi Arabia and Jordan was low despite the high prevalence of Covid-19 [30]. According to a study conducted in Ethiopia, the general tendency to receive the vaccine was low [2, 28]. Low levels of trust in government, low or moderate Covid-19 mortality, low level of education, low income, unemployment and old age were all factors contributing to the low willingness to receive the vaccine [31]. Shekhar et al. In the United States found that 36% of respondents were willing to receive the vaccine as soon as it became available, while 56% were reported unsure or waiting for further information.

According to the above, the percentage of vaccinations in different countries or regions is not only affected by the supply side, but also by the demand side. In other words, even if a country provides full access to vaccination for all people, a percentage of people still do not want to be vaccinated. Therefore, a raw comparison of the percentage of vaccinations may not reflect this fact. However, the aim of the researchers was only to provide an overview of inequality in vaccination coverage (whether affected by the supply side or affected by the demand side) and the factors affecting it. At the same time, in future researches, it can be examined to what extent the lack of vaccination coverage is related to the lack of access and to what extent is related to the unwillingness to receive it.

Due to the importance of vaccine distribution and its impact on human health and the world economy, the present review study was conducted to determine inequality in vaccine distribution in the world. The results of the study can provide valuable information to health policy makers, especially globally.

## Methods

The review protocol was registered on Prospero (PROSPERO acknowledgement of receipt [338851]). The systematic review was conducted and reported in accordance to the PRISMA guidelines (http://www.prisma-statement.org/). In the first step, the research question is determined based on the elements of PCC (population, concept and context). In the present study, identifying the inequality of Covid-19 vaccine distribution and the factors affecting it (concept) in all countries of the world (population) in which vaccination has been performed (context), has been raised as a key question.

### Search strategy and data sources

During the second step, search phase, the target population was all studies related to the distribution of vaccines in different countries of the world. For this purpose, all relevant studies from December 2020 to the 30 May, 2022 were retrieved through a detailed research strategy (Table 1).

**Table 1:** The search strategy of the study

Database	Search string	Number of retrieved papers	Limits
**Scopus**	(TITLE-ABS-KEY (“covid19” OR corona OR “covid-19” OR “SARS-CoV-2”)) AND (TITLE-ABS-KEY (vaccine OR vaccination OR immunization OR immunisation)) AND (TITLE-ABS KEY (inequality OR inequity OR disparity OR distribution)) AND (LIMIT-TO (PUBYEAR, 2021) OR LIMIT-TO (PUBYEAR, 2020)) AND (LIMIT-TO (DOCTYPE, “ar”) OR LIMIT-TO (DOCTYPE, “re”)) AND (LIMIT-TO (LANGUAGE, “English”)) AND (LIMIT-TO (SRCTYPE, “j”))	2389	Language (only resources with at least an abstract in English), search the key words in title and abstract. Date: December 2020 (the first vaccination), up to 30 May, 2022
**PubMed**	Search: ((“covid19”[Title/Abstract] OR corona*[Title/Abstract] OR “covid-19”[Title/Abstract] OR “SARS-CoV-2”[Title/Abstract]) AND (vaccin*[Title/Abstract] OR immunization [Title/Abstract] OR immunization [Title/Abstract])) AND (inequality [Title/Abstract] OR inequity [Title/Abstract] OR disparity [Title/Abstract] OR distribution [Title/Abstract]) Filters: English, from 2020 to 2022	1125	
**WOS**	TI = ((“covid19” OR corona OR “covid-19” OR “SARS-CoV-2”) AND (vaccine OR immunization OR immunisation OR vaccination) AND (inequality OR inequity OR disparity OR distribution)) AB = ((“covid19” OR corona OR “covid-19” OR “SARS-CoV-2”) AND (vaccine OR immunization OR immunisation OR vaccination) AND (inequality OR inequity OR disparity OR distribution))	916	
**ProQuest**	TiAb(“covid19” OR corona OR “covid-19” OR “SARS-CoV-2”) AND ab (vaccine OR immunization OR immunisation OR vaccination) AND ab (inequality OR inequity OR disparity OR distribution)	193	

Research keywords include (“covid19” OR corona OR “covid-19” OR “SARS-CoV-2”) AND (vaccine OR vaccination OR immunization OR immunisation) AND (inequality OR inequity OR disparity OR distribution), Which were searched in PubMed, Web of Science, Scopus and ProQuest databases within the time frame mentioned.

### Inclusion and exclusion criteria

Inclusion criteria were articles with at least one English abstract indexed in one of the mentioned databases, which pointed to inequality in vaccine distribution based on selected keywords and their synonyms. Also, letter to editor, commentary and types of reviews and those studies that had not been published by the time of the study were considered as exclusion criteria.

**Fig. 1:**
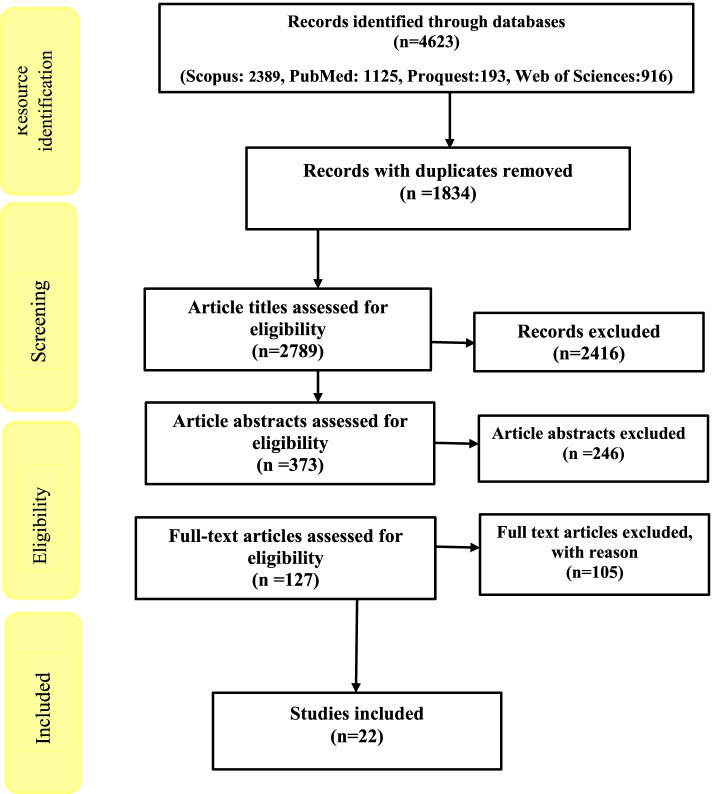
PRISMA flow diagram of searching and selection procedure for inequality in the distribution of Covid-19 vaccine

In the third step, the indexed information of the studies in the mentioned databases was transferred to the EndNote software with the help of keywords and the relevant studies were selected according to the purpose of the research. In the first stage, the selection was done with the help of the research title and in the next stages, this selection was done using the abstract and reading the full text of the articles, respectively. It should be noted that all stages of research and selection of studies have been done by two researchers independently (F.S.J. and M.Q.) and if necessary, the third researcher was asked to help reach a consensus (M.B) (Fig. 1).

### Quality assessment of articles

Qualitative evaluation, in addition to selection of related articles and extraction of their data were performed by two researchers separately. Selected articles were qualitatively evaluated by researchers using the STROBE (Strengthening the Reporting of Observational studies in Epidemiology) checklist (https://www.strobe-statement.org/).

Any disagreement was referred to a third party by two evaluators. This checklist consists of 22 different sections and evaluates various aspects of methodology including sampling methods, measurement of variables, statistical analysis, adjustment of confounders, mentioning the validity and reliability of the tools used and the objectives of the study. The final quality score of articles based on this tool is reported in the last column of the attached table (Additional file 1: Appendix –Table 1).

## Results

The results of searching articles in databases showed that there were 4623 articles in the field of study purpose, of which 1834 articles were duplicates. Then 2416 articles in terms of title, 246 articles in terms of abstract and 105 articles in terms of full text of articles that did not meet the inclusion criteria were rejected. Finally, 22 articles were selected and entered into the study.

Findings from the descriptive analysis of 22 studies showed that 15 (68.17%) studies were conducted by researchers in the United States, three (13.64%) by Europe, three (13.64%) by Asia and one by Oceania (4.55%) authors. Also, 18 (81.81%) of the studies were related to developed countries and four of them (18.19%) was related to developing countries. Detailed information of included studies were summarized in the additional file, Additional file 1: Appendix (Table 1).

Based on the thematic analysis of the findings, the researchers categorized the factors explaining the inequality in the distribution of COVID-19 vaccine into macro and micro levels Table 2.

**Table 2:** Factors affecting inequality in the distribution of Covid-19 vaccine

Main categories	Subcategories	Factors	References
**Macro level (country)**	Economic	Stability and country’s economic status	[32–35]
		GDP per capita	[35–38]
		Financial support	[33, 36]
		Human development index	[35, 36]
	Infrastructure and health system	Appropriate information system	[33]
		Functional cold chains in vaccine Transport	[33]
		Transport infrastructure	[33]
		Medical and non-medical facilities per capita	[32, 37]
		Healthcare provision and access	[32, 33]
		Healthcare quality	[32]
	Legal and politics	Vaccination allocation rules	[33]
		Health policies	[37]
		Political ideology	[32]
		Racial bias	[32]
	Epidemiologic and Demographic	Covid-19 incidence rate	[32, 36, 39]
		Covid-19 deaths rate	[36, 40]
		Life expectancy	[36]
		Vulnerability to covid-19	[36]
		Working in medical setting	[40]
		Comorbidities	[40]
		Social vulnerability	[41]
		Incarceration index	[34]
		Education index	[32, 34, 42]
**Micro level (individual)**	Economic characteristics	Household’s income	[33, 39, 40, 42–46]
		Home ownership	[39]
		Employment	[34, 39, 43]
		Poverty	[36, 43, 47]
		Access to healthy food	[39]
		Residency in the deprived areas	[45, 47–49]
	Demographic and social characteristics	Sex	[39, 49]
		Age	[34, 36, 39, 40, 42, 44–46, 48, 50, 51]
		Race	[32, 34, 40–44, 48, 51–53]
		Ethnic	[41, 44, 45, 48, 49, 52, 53]
		Religion	[48]
		Disability	[48]
		Location (urban/rural)	[39, 46, 49]
		Insurance coverage	[39]

Significant to mention is, if the factors explain the inequality of vaccination rates between different countries or regions (for example, states), they are classified as macro level factors. In other words, aggregate indices have been used to explain inequality. For instance, countries with higher per capita incomes reported higher coverage rates. On the other hand, if they explain inequality factors in vaccination between individuals or households in a country or region, they were classified as micro level factors. This means that variables have been reported at the individual level to explain inequality. For example, men have received more vaccinations than women.

### Micro (individual) level factors

According to the results of the present study, the most influential factors on vaccine distribution were related to micro level (individual) factors. Demographic and social characteristics at the micro level were the most important factors influencing the inequality of the distribution of covid-19 vaccine. These components are Economic characteristics (household’s income, home ownership, employment, poverty, access to healthy food, residency in the deprived areas) and Demographic and social characteristics (sex, age, race, ethnic, Religion, disability, Location (urban/rural), insurance coverage).

Nineteen studies pointed to the factors affecting inequality in the distribution of COVID-19 vaccine at the micro level. Factors affecting the individual level were classified into two groups including economic characteristics and demographic and social characteristics. Among these, thirteen studies (68.42%) referred to the factors of economic characteristics and all studies referred to the factors of demographic and social characteristics of individuals.

### Macro (country) level factors

According to the findings, 11 studies examined the factors affecting the equitable distribution of COVID-19 vaccine at the macro level. Factors affecting the distribution of COVID-19 vaccine at the macro level were divided into four groups, economic (Stability and country’s economic status, GDP per capita, Financial support, Human development index), infrastructure and health system (Appropriate information system, Functional cold chains in vaccine Transport, Transport infrastructure, Medical and non-medical facilities per capita, Healthcare provision and access, Healthcare quality), legal and politics (Vaccination allocation rules, health policies, Political ideology, racial bias), epidemiologic and demographic (Covid-19 incidence rate, Covid-19 deaths rate, Life expectancy, Vulnerability to covid-19, Working in medical setting, Comorbidities, Incarceration index and Education index). Seven studies on economic factors (63.63%), three studies on infrastructure and health system factors (27.27%), three studies on legal and political factors (27.27%), and seven studies on demographic and epidemiological factors (63.63%) are focused.

## Discussion

Inequality in the distribution of the Covid-19 vaccine is one of the major challenges in managing the corona pandemic internationally and nationally. There are different legal, economic, social and demographic factors in the Covid-19 vaccine distribution in countries that have disrupted the process of fair vaccination. The aim of this study was to identify the factors affecting the distribution of Covid-19 vaccination.

Demographic and social characteristics factors at the micro level were among the most frequent components affecting the distribution of Covid-19 vaccine. For example, according to some studies in the United States and the United Kingdom [39, 49], the rate of vaccine injection is higher in women than men. Also, older people had greater access to vaccination [34, 36, 39, 40, 42, 44, 48, 50, 51]. A study by Cardona et al. in Maryland found that vaccination rates were lower in blacks [32]. Villagers have received fewer vaccinations than urban dwellers [39, 46, 49]. Vaccination rates were higher in people covered by insurance [39]. This indicates that in some countries, financial access has affected vaccine eligibility. A study by Vahe et al. in the United Kingdom found that among religious groups, the highest distribution of the vaccine was among Christians and the lowest among Buddhists. Vaccination rates have also been lower in people with severe disabilities [48].

Another individual factor creating inequality in Covid-19 vaccination was economic characteristics. According to many studies, low-income individuals or households [33, 39, 40, 42–44], were less likely to receive the Covid-19 vaccine. According to a study by Donadio et al. in the United States, homeless people were less likely to be vaccinated [39]. There was a low negative correlation between the unemployment rate and the percentage of vaccinated people. In other words, people who were unemployed had a lower vaccination rate [39]. Other economic factors affecting the lack of access to Covid-19 vaccine were poverty [36, 43] and living in deprived areas [45, 47–49]. Vaccination rates were also lower in people with limited access to healthy food [39]. Therefore, it seems that the low economic situation has been one of the most important factors in reducing the availability of Covid-19 vaccine.

The most common macro-level factors were epidemiologic and demographic factors. According to a study in California, vaccination rates were higher in areas with socially disadvantaged populations [41]. Countries with the most vulnerable groups to Covid-19 had priority on access to vaccination, higher doses, and thus better vaccination coverage [36]. Another study found that vaccination rates were higher in countries with higher life expectancy [36]. Also, in countries with a higher incidence and mortality from Covid-19, the vaccination rate was higher [36, 40]. Vaccination rates were higher in health care workers and those with significant comorbidities [40]. According to a study, the rate of Covid-19 vaccination is higher among US prisoners than in other groups [34]. Covid-19 vaccination rates have also been reported in countries with higher education [42]. Seemingly the selection of vaccine distribution criteria based on the vulnerability of people in the community is one of the common methods of vaccine distribution. Because by doing so, people prone to Covid-19 disease will be vaccinated sooner and the virus transmission chain will be cut faster.

Infrastructure and health system factor is one of the components affecting the fair distribution of the vaccine at macro level. A study by Duan et al. in China showed that the lack of appropriate information systems, cold chains used in vaccine delivery, and transportation infrastructure in countries have affected the rapid spread of vaccines [33]. Covid-19 vaccination rates were also lower in areas with low access to health facilities and poor quality of health care [32]. It can be concluded that in countries/areas where there are adequate health facilities and infrastructure for the production and distribution of Covid-19 vaccine, access to Covid-19 vaccine is higher.

Another macro factor influencing vaccine distribution was the legal and politics factor. Agarwal in the United States [32] showed that the results of the 2020 presidential election, followed by the prevailing political opinion, influenced the distribution of the Covid-19 vaccine. According to the WHO, the implementation of unjust health policies in the field of vaccine distribution has put the world on the verge of a catastrophic moral failure, and the cost of this failure is mostly paid by poor countries with the lives of people [37]. According to a study by Duan et al. in China, the lack of sustainable vaccination management [33] affects the fair distribution of vaccines. Racial prejudice is also another factor in the unequal distribution of the Covid-19 vaccine. According to a study, politicians’ racial bias has led to whites and indigenous peoples in the region receiving a higher percentage of vaccines [32]. Existence of fair laws and policies in situations where the collective interests of countries are at stake can reduce the rapid transfer and mortality of Covid-19. Therefore, in countries where the distribution of vaccines has been in accordance with clear principles and rules and far from wrong policies, the vaccination rate against Covid-19 has been higher.

Macro-economic factors were other factors influencing the distribution of the Covid-19 vaccine. According to a study, in low and middle-income countries, lower coverage rates of Covid-19 vaccination have been reported [32, 33]. In the study by Roghani et al. higher per capita GDP is positively correlated with greater distribution of Covid-19 vaccine [37]. Financial aid in countries was one of the main determinants that enabled the faster spread of vaccination. Countries such as the US, China, UK, and India, which have the highest rates of human development, have also reported higher percentages of vaccinations [36]. It can be concluded that at the time of pandemics, the economic level and income of countries are important factors in providing appropriate preventive services, including vaccination coverage to combat the disease.

## Conclusion

According to our systematic review, there were inequality in COVID-19 vaccine distribution among different countries, areas or people. Factors affecting these inequalities were classified to micro and macro level determinants. Four categories of micro level factors include economic, infrastructure and health system, legal and politics, and epidemiologic and demographic factors. Plus, factors such as economic characteristics, in addition to demographic and social characteristics came up at the macro level. At macro level factors, variables such as GDP per capita, stability and country’s economic status, Covid-19 incidence rate, education index, financial support, human development index, medical and non-medical facilities per capita, healthcare provision and access, and Covid-19 deaths rate had the highest frequency in researches. Among micro level determinants, age, race, ethnic, household income, residency in the deprived areas, employment, poverty, location (urban/ rural) and gender were most often mentioned in the literature. Findings showed that factors in different levels have had effects in the inequality of COVID-19 vaccine distribution. Thus health policy makers in all levels of global, regional, national and local must plan and take action in the reduction of inequality in COVID-19 vaccine distribution.

### Limitation

Among the limitations of this study are the limitation of search databases by researchers and the possibility of inaccurate vaccination rate statistics in various studies.

The current study provides sound evidence on factors affecting Covid-19 vaccine distribution at micro and macro level. These evidences can help policy makers at international, national and local level to reduce inequality in distribution of Covid-19 vaccine.

## Supplementary Information

**Additional file 1: Appendix Table 1.** Detailed information of selected studies.

## Data Availability

Data of this research is available and could be sent upon contact with the corresponding author.
