# Supplementary material for: Inequality in the distribution of Covid-19 vaccine: a systematic review
Source: Int J Equity Health. 2022 Aug 30;21:122. doi: 10.1186/s12939-022-01729-x (PMC9425802; doi:10.1186/s12939-022-01729-x)
Supplement: Supplementary file 1 — Additional file 1: Appendix Table 1. Detailed information of selected studies. [file 12939_2022_1729_MOESM1_ESM.docx]

**Appendix:**

**Table 1. Detailed information of selected studies**

| **Quality assessment score** | **Results** | **Analysis** | **Participants** | **Place** | **Year** | **First Author** | **Title** | **N** |
| --- | --- | --- | --- | --- | --- | --- | --- | --- |
| 17 | Black and Latino clustered are less likely to have vaccine distribution sites and also negatively related to the number of allocated vaccine doses (racial-ethnic inequality) | series of spatial error and linear growth models | five largest urban counties in Texas | United states | 2022 | [Kathryn Freeman](https://journals.sagepub.com/action/doSearch?target=default&ContribAuthorStored=Anderson%2C+Kathryn+Freeman) | Racial-Ethnic Residential Clustering and Early COVID-19 Vaccine Allocations in Five Urban Texas Counties (54) | 1 |
| 16 | There was a strong linear association between per capita income and the proportion of people vaccinated | Data analysis and visualization were performed in R-Studio (Linier regression) | 153 countries with one million or more population | UK | 2022 | Palash Basak | A Global Study on the Correlates of Gross Domestic Product (GDP) and COVID-19 Vaccine Distribution (40) | 2 |
| 16 | the median per capita income, human development index, and health expenditure per capita. | estimated the vaccination uptake rate across countries by fitting a logistic model to reported daily case numbers.  used Random Forest, to study the association between vaccination uptake rate and socio-economic factors | 142 countries | Canada | 2022 | Kazemi | Assessing Inequities in COVID-19 Vaccine Roll-Out Strategy Programs: A Cross-Country Study Using a Machine Learning Approach (37) | 3 |
| 15 | Demographics, socioeconomic factors, and experiencing economic hardship during the pandemic each explained a statistically significant portion of vaccination coverage disparities between non Hispanic White and racial/ethnic minority individuals. | a regression-based decomposition method was used to estimate how much of the observed racial and ethnic disparities in vaccination coverage could be explained by particular socioeconomic and demographic factors | 340,046 household respondents | United states | 2022 | Williams | Racial and Ethnic Disparities in COVID-19 Vaccination Coverage: The Contribution of Socioeconomic and Demographic Factors (44) | 4 |
| 17 | Following socioeconomic variables were related to vaccine coverage:  -Insurance coverage (coefficient (95% ci) −3.3 (−3.6, −3.0), housing (0.8 (0.5, 1.2)), income (−1.2 (−1.6, −0.9)), unemployment level (−0.7 (−1.0, −0.3)), rural (−3.8 (−4.2, −3.5)), black (−1.7 (−2.0, −1.3)), limited access to healthy food (−1.2 (−1.5, −0.8)), sex p-value <0.001, homeownership (−0.5 (−0.9, −1.6)), above 65 years of age (0.062, p-value <0.001). | Correlations were computed between cumulative vaccination rate and change in covid-19 incidence from 1 December 2020 to 6 June 2021, with 44 different demographic, environmental, and socioeconomic factors. Multivariate linear regression was also used to adjust for age as a potential confounding variable. | 328 million individuals from all 50 states | United states | 2021 | Gregory Donadio | Counties with lower insurance coverage and housing problems are associated with both slower vaccine rollout and higher covid-19 incidence (41) | 5 |
| 16 | Social determinants affecting covid-19 vaccination disparities were:  -Economic stability including median income and median income disparity; P-value< 0.05  -Education access and quality (high school disparity); P-value<0.01  -Healthcare access and quality including health facilities per capita and covid-19 cases per capita; P-value< 0.05  -Social and community context including rate of vehicle ownership, political ideology, segregation index and racial bias; P-value< 0.01 | Covid-19 vaccination data were collected from state public health department websites. Weighted OLS regression analysis was used for factors affecting on Covid-19 vaccination. | 756 US counties, covering over 170.6 million  people | United states | 2021 | Ritu Agarwal | Socioeconomic privilege and political ideology are associated with racial disparity in covid-19 vaccination (44) | 6 |
| 15 | Determinants of vaccine doses per thousand were GDP per capita (positive), extreme poverty (negative), life expectancy (positive), and HDI (direct); P-value<0.01.  Moreover, extreme poverty (negative direction), life expectancy, and median age were associated with days of vaccination (P-value<0.01) | The structural equation modeling was used to assess relationship between the country's socioeconomic variables with covid-19 vaccination rate. Moreover, confirmatory factor analysis, t-test, and Pearson’s correlation were used. | 189 countries | Brazil | 2021 | Brigitte Renata | Determinants of access to the sars-cov-2 vaccine: a preliminary approach (38) | 7 |
| 18 | Vaccination disparity was ranged from 0.14 to 1.82 among counties.  Race, median household income, Covid-19 cases and deaths and age were among the factors affecting vaccination disparity among Maryland counties. | Descriptive statistics and risk ratio analysis were used to measure the association between vaccination disparity and other factors. | 10 Maryland counties with the highest incidence of covid-19 mortality and morbidity | Maryland | 2021 | Stephanie Cardona | Vaccination disparity: quantifying racial inequity in covid-19 vaccine administration in Maryland (42) | 8 |
| 16 | Upper-middle (β = -1.44, P <0.001), lower-middle (β = -2.24, P< 0.001), and low (β = -4.05, P<0.001) income countries had lower vaccination coverage compared with high-income countries.  14.6% and 15.6% of the effect in upper-middle and lower-middle income countries were mediated by vaccination policies. | Descriptive statistics, single mediator model based on structural equation modeling for different income groups, and linear regression models were used to assess inequality in average vaccination coverage among countries. | 138 countries | China | 2021 | Yuqi Duan | Disparities in covid-19 vaccination among low-, middle-, and high-income countries: the mediating role of vaccination policy (35) | 9 |
| 17 | GDP per capita (odds ratio: 1.38, p value<0.01), health policies (p value<0.01) medical (Hospital bed) and non-medical facilities distribution (odds ratio: 1.23, p value<0.01) were macro determinants of vaccination rate. | Descriptive statistics, correlation and regression analysis were used to analysis relationship between vaccination rate with other socioeconomic factors. | 25 countries | United states | 2021 | Ali Roghani | The global distribution of covid-19 vaccine: the role of macro-socioeconomics measures (39) | 10 |
| 15 | Distribution of Covid-19 vaccination was based on age groups. | Descriptive analysis | Ontario population | Canada | 2021 | [Gareth](https://www.ncbi.nlm.nih.gov/pubmed/?term=Leung%20G%5BAuthor%5D&cauthor=true&cauthor_uid=34367770) Leung | Epidemiological study of covid-19 fatalities and vaccine uptake: insight from a public health database in Ontario, Canada (52) | 11 |
| 18 | There were disparity in Covid-19 vaccination coverage between minority groups (black, Hispanic, and Asian).  Demographic (age and household size), socioeconomic factors  (health insurance, education, income and employment)  And experiencing economic hardship during the pandemic were the most important determinants of vaccine coverage distribution (p-value<0.01) | Description and regression based decomposition approach was used. | Adults from the US census bureau household pulse survey | Georgia | 2021 | Austin M Williams | Racial and ethnic disparities in covid-19 vaccination coverage: the contribution of socioeconomic and demographic factors (55) | 12 |
| 17 | In counties with lower income inequality, a 1% increase in poverty rate associate with a 3.5% (95% CI 1.5% and 5.5%) decrease in the odds of fully vaccination.  In county with higher income inequality, a 1% increase in a black population corresponds to a 1.3% (95% CI 0.2% and 2.8%) decrease in the odds of being fully vaccinated. This figure was 2.6% (95% CI 1.4% and 3.8%) for Hispanic population. | Fractional logit model was used for assessing the countys’ socioeconomic factors and vaccination rate. | 102 counties in the Illinois state | United states | 2021 | [Tim F Liao](https://www.nature.com/articles/s41598-021-97705-6#auth-Tim_F_-Liao) | Social and economic inequality in coronavirus disease 2019 vaccination coverage across Illinois counties (45) | 13 |
| 16 | Lower vaccination rates were found among all minority ethnic groups (87.7%) compared with the white people (94.0%).  Muslim (79.1%) and Buddhist (84.1%) have lower rate of vaccination.  Younger age, lower area deprivation, more advantaged socioeconomic status, not being disabled were correlated with higher likelihoods of receiving the vaccine (P<0.05). | Logistic regression analysis was used to estimate unadjusted and adjusted odds ratios for vaccination rate based on socio-demographic factors. | 6655672 adults aged ≥70 years | England | 2021 | Vahe Nafilyan | Sociodemographic inequality in covid-19 vaccination coverage among elderly adults in England: a national linked data study (50) | 14 |
| 19 | The largest inequality was disparity among ethnic group (white: 94.1% VS black: 73.9%, P-value<0.01)  There was also significant inequality by gender (male: 90.8%, female: 93.4%, P-value<0.01) and rural/urban residency (rural: 92.5%, urban: 91.9% P-value<0.01). Moreover, residents in the most deprived areas had lower chance of being vaccinated (P-value). | Odds ratios of being vaccinated were estimated using univariable and multivariable logistic regression models. | 1256412 individuals aged 50 years and over | UK | 2021 | Malorie Perry | Inequalities in coverage of covid-19 vaccination: a population register based cross-sectional study in wales, UK (51) | 15 |
| 17 | The results of simulated adjusted COVID-19 vaccine equity index for non-Hispanic white (0.66), black/African American (2), Asian (1.06), and Hispanic (2.84) showed that vaccination rate in black and Hispanic population should be increased in order to achieve equity in vaccine distribution. | Simulation approach was performed to estimate Covid-19 vaccine equity index (CVEI). Using Conditional probability, equity for unvaccinated individuals and CVEI were estimated for specific subgroups. | - | United states | 2021 | Alice R Pressman | Measuring and promoting sars-cov-2 vaccine equity: development of a covid-19 vaccine equity index (53) | 16 |
| 16 | Higher vaccine availability was found in areas where the population was older, richer, and whiter.  There was strong relationship between the number of vaccination sites with race and ethnicity, and weaker association with population percentage at or below the poverty line. | Hotspot and multivariate analysis was done to measure spatial clustering of Publix vaccination sites per 100,000 people. | 974 households | Florida | 2021 | [Attonito](https://pubmed.ncbi.nlm.nih.gov/?sort=pubdate&size=200&term=Attonito+J&cauthor_id=34871075) | Sociodemographic disparities in access to covid-19 vaccines upon initial rollout in Florida (46) | 17 |
| 15 | After using the developed tools, in patients higher 65 years of age, significant improvements in vaccinating more vulnerable populations, Black and Hispanic were seen (P-value<0.001) | An interactive online tools was created to measure equitability of vaccine distribution and to identify populations groups eligible for Covid-19 vaccine. | All UNC community vaccinations | North Carolina | 2021 | Shaheen | Interactive, on-line visualization tools to measure and drive equity in covid-19 vaccine administrations (43) | 18 |
| 18 | Analytic results showed following factors effect on inequality between COVID-19 vaccination rates across 45 states.  -Hispanic state racism index (−2.87, −0.84 to−4.90 (95%CI))  -Economic index (−2.46, −4.63 to−0.29 (95%CI))  -Employment index ( −1.47, −3.42 to+0.48 (95%CI))  -Education index (−2.73, −5.45 to+0.00 (95%CI))  -Incarceration index (−2.98, −0.82 to−5.14 (95%CI)) | Descriptive and regression analyses of determinants of disparities in COVID-19 vaccination rates across 45 states. | Population in all states | United states | 2021 | Michael Siegel | Racial/ethnic disparities in state‑level covid‑19 vaccination rates and their association with structural racism (36) | 19 |
| 17 | Older age and higher socioeconomic status (SES) were associated, with stepwise higher cumulative vaccination rates.  lowest vaccination rates in Arab (lower coronavirus vaccination coverage among minority and disadvantaged groups) | estimated vaccination hazard and cumulative incidence using the Fine and Gray competing risk model | 6 478 999 individuals age 15 years and no COVID-19 history | Israel | 2022 | Benderly | Fighting a pandemic: sociodemographic disparities and coronavirus disease-2019 vaccination gaps—a population study (47) | 20 |
| 16 | The periphery and poor areas of the city had the least access to COVID-19 vaccination centers. | Global Moran’s index (GMI) was used to measure the spatial autocorrelation of the accessibility index in different scenarios and the proposed model. | 26 public hospitals and 271 public healthcare centers | Iran | 2021 | Mohammadi | Measuring COVID-19 vaccination coverage: an enhanced age-adjusted two-step floating catchment area model (49) | 21 |
| 18 | Spatially accessibility to vaccination services varies, and be better in major cities than rural regions.  priority populations including older people, people living in rural areas and residents of areas with socioeconomic constraint have, on average, statistically significantly lower spatial access to vaccination services. | used the enhanced two-step-floating-catchment-method (E2SFCA) to estimate spatial access to vaccination services | Aotearoa’s population | New Zealand | 2021 | Whitehead | Structural disadvantage for priority populations: the spatial inequity of COVID-19 vaccination services in Aotearoa (48) | 22 |
